# Targeting Toll-Like Receptors for Treatment of SLE

**DOI:** 10.1155/2010/498980

**Published:** 2010-09-19

**Authors:** Christopher G. Horton, Zi-jian Pan, A. Darise Farris

**Affiliations:** ^1^Department of Microbiology and Immunology, University of Oklahoma Health Sciences Center, Oklahoma City, OK 73104, USA; ^2^Arthritis and Immunology Program, Oklahoma Medical Research Foundation, 825 NE 13th Street, Oklahoma City, OK 73104, USA

## Abstract

Toll-like receptors (TLRs) are important innate immune receptors for the identification and clearance of invading pathogens. Twelve TLRs that recognize various conserved components of microorganisms are currently known. Among these, the endosomal TLRs 3, 7/8, and 9 recognize dsRNA, ssRNA, and CpG DNA, respectively. Nucleic acid-sensing TLRs, TLR 7 in particular, have been implicated in systemic lupus erythematosus (SLE) and are thought to exacerbate disease pathology. Activation of these TLRs results in the production of inflammatory cytokines and type I interferon. Genome-wide association studies, single nucleotide polymorphism analyses as well as experimental mouse models have provided evidence of TLR signaling involvement in SLE and other autoimmune diseases. Since activation of these receptor pathways promotes autoimmune phenotypes, inhibitory drugs that target these pathways constitute important new therapeutic strategies for the treatment of systemic autoimmunity.

## 1. Introduction

Toll-like receptors (TLRs) are pattern recognition receptors of the innate immune system that recognize specific pathogen-associated molecular patterns (PAMPs) conserved among microorganisms. There are currently twelve known TLRs in mammals, which identify common constituents of invading pathogens including double-stranded and single-stranded RNA, unmethylated CpG DNA, bacterial lipopolysaccharide (LPS), lipoproteins, and flagellin [1]. Upon interaction with their ligands, TLRs signal through adapter proteins, most commonly Myeloid Differentiation Primary Response Gene 88 (MyD88), though TLR 3 alternatively signals via the TIR-Domain Containing Adaptor Inducing Interferon-*β* (TRIF) adapter [2]. These adapters recruit other molecules to initiate signaling cascades ultimately leading to the production of proinflammatory cytokines and type I IFN [3]. These TLR responses are important in the functioning of both the innate and adaptive arms of immunity [4, 5]. 

 Although TLRs are important for antimicrobial immunity, they have also been implicated in the pathogenesis of autoimmune diseases. For example, TLRs 2 and 4 have been identified as factors involved in the onset of type I diabetes mellitus [6–9], and TLRs 1–6 are expressed by rheumatoid arthritis (RA) synovial fibroblasts and are thought to provoke joint inflammation in RA [10, 11]. Moreover, the nucleic acid binding TLRs 7 and 9 have been connected to both human and mouse models of systemic lupus erythematosus (SLE) [12–17]. Because of these links between TLRs and autoimmunity, a great deal of work has been directed toward understanding how these receptors act in disease progression. Two major possibilities arise in describing how TLRs might work in autoimmunity; either they are stimulated by exogenous antigens, like viral ssRNA, which then stimulate resident immune cells, or the TLRs recognize endogenous self-antigens to initiate and propagate inflammation and autoimmunity. 

The accumulation of evidence pointing towards TLRs in autoimmunity has opened the door for potential therapeutic interventions directed towards the modulation of Toll-like receptors and their signaling pathways. Since TLRs are normally responsive to microbial pathogens, there has been some speculation as to the application of TLR agonists as vaccine adjuvants to stimulate more robust immune responses [18, 19]. On the other side of the spectrum, for autoimmunity, disease can result from aberrant hyperactive signaling; therefore, the application of inhibitory, or antagonistic, TLR therapeutics is of considerable interest. A few inhibitors have been developed already and show some promise for potential human therapeutics. The focus of this paper is to summarize the present literature documenting how modulation of Toll-like receptor pathways may be used as potential methods of treatment for autoimmunity, particularly SLE.

## 2. TLR Function and Signaling

TLRs are localized to either the cell surface or endosomes of several cell types, most notably of antigen-presenting cells (APCs) such as dendritic cells [20, 21] and B cells [22]. Under normal circumstances, TLRs aid in the identification and removal of materials that may be detrimental to the host organism; these are usually of bacterial, viral, fungal, or protozoan origin [1, 23]. Because TLRs recognize common molecular motifs instead of specific peptide sequences, they maintain capacity for recognition of a broad repertoire of microbes. We know, with relative certainty, what most of the TLRs recognize and that the end result is inflammation, but how does this occur? The cascade by which TLRs induce an inflammatory environment varies depending on the particular TLR that is stimulated. Since we are more concerned with SLE, we will concentrate on the signaling that takes place upon activation of nucleic acid-binding TLRs 3, 7/8, and 9. 

**Figure 1:**
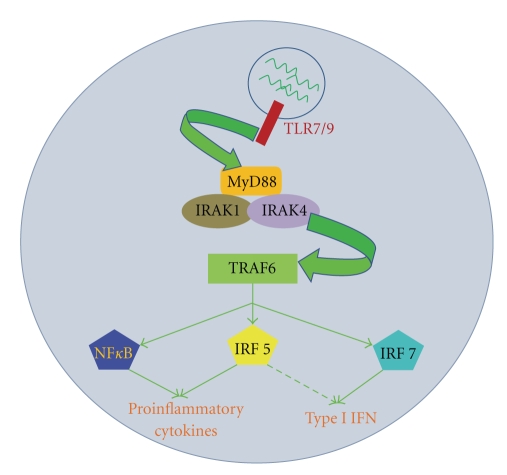
TLR7/9 Signaling pathway leading to the production of proinflammatory cytokines and type I IFN. Receptor recognizes nucleic acid ligand and recruits adaptor protein MyD88. MyD88 attracts kinases IRAK1 and IRAK4 to the complex, which can then activate TRAF6. Activated TRAF6 leads to the stimulation of transcription factors NF*κ*B, IRF5, and IRF7 to produce cytokines and type I IFN. IRF7 has been identified as the primary factor in production of type I IFN, but there is evidence that IRF5 can also contribute to type I IFN production [36].

TLRs exist as dimers or heterodimers with the capacity to engage their respective ligands. Ligand binding is thought to induce a conformational change resulting in interaction or close juxtaposition of the two cytosolic Toll/IL-1 receptor (TIR) domains, thus providing an interface for adaptor protein binding and subsequent signal transduction [24]. As described by several groups, MyD88 is the most common of these adaptors and has been shown to be involved in signaling through all TLRs except TLR 3 [24–27]. This adapter protein provides a scaffold for further interaction with IL-1R-associated kinase (IRAK) 1 and 4 to propagate the signal to downstream effectors via phosphorylation. Once recruited to the adaptor protein, IRAK4 is activated. IRAK4, in turn, activates IRAK1 via phosphorylation [28]. These activated kinases recruit tumor necrosis factor receptor-associated factor (TRAF) 6, which is an E3 ubiquitin ligase required for activation of NF*κ*B by freeing it from its inhibitor, I*κ*B [29]. In addition to this, interferon regulatory factors (IRFs) 5 and 7 are also recruited to the MyD88/IRAK/TRAF6 complex, where they can become phosphorylated and activated [30]. Ultimately, the activation of transcription factors NF*κ*B and IRFs 5 and 7 results in their translocation into the nucleus where they initiate gene transcription and production of proinflammatory cytokines and type I IFN (Figure 1) [3, 31–35]. 

TLR 3 signaling is distinct from the previously described TLR 7 and 9 signaling pathways. Instead of utilizing MyD88 as an adaptor protein, TLR 3 uses TRIF [2]. As with MyD88, TRIF also recruits additional proteins necessary for downstream signaling, including TRAF-family-member-associated NF*κ*B-activator-binding kinase 1 (TBK1), TRAF6, and receptor-interacting protein 1 (RIP1) [24]. TRIF interaction with TBK1 is necessary for the activation of IRF3, which is a transcription factor involved in the production of IFN-*β* [37]. TLR 3 can also activate NF*κ*B by the interaction of TRIF with TRAF6 or RIP1, which allow the transcription factor to migrate to the nucleus to induce target gene transcription [24, 28]. 

The multiple proteins involved in TLR signaling yield many opportunities to inhibit this process. Several endogenous inhibitors have been identified that halt, or at least impair, the signaling cascade, dampening the TLR-mediated production of inflammatory cytokines and type I IFN. The ability to modulate TLR signaling helps to maintain a homeostatic balance important for antimicrobial immunity while also preventing collateral damage to self-tissues. The inhibitory proteins important in this process target the receptors themselves, adapter molecules, and key kinases, as well as transcription factors to diminish the TLR-mediated production of inflammatory cytokines and type I IFN. Triad3A acts as an E3 ubiquitin ligase promoting the degradation of TLR 4 and 9. The degradation of these TLRs was shown to impair NF*κ*B activity upon stimulation with LPS and CpG DNA whereas siRNA knockdown of Triad3A enhanced LPS and CpG DNA-mediated NF*κ*B activity [38] indicating a prominent role of Triad3A in modulating TLR response. Although Triad3A appears to act specifically on TLR 4 and 9, other inhibitors provide a more global impact on TLR signaling. As mentioned earlier, MyD88 is an adapter protein downstream of all TLRs except TLR 3. Upon stimulation with LPS or TNF, a splice-variant of MyD88, MyD88s, can be induced which prevents activation of NF*κ*B [39]. This short form of MyD88 fails to recruit IRAK4, impairing the ability to phosphorylate IRAK1, therefore preventing the activation of NF*κ*B [39], although maintaining the ability to activate the transcription factor AP-1 [40]. In addition, recently, An et al. found that the phosphatase, SHP-1, selectively impairs activation of NF*κ*B and subsequent production of proinflammatory cytokines while also inhibiting IRAK1 resulting in an increase in IFN-*β* [41]. Several other factors have inhibitory roles directed to the active IRAK kinases. Kobayashi et al. showed that IRAK-M, which lacks the kinase activity of its counterparts IRAK1 and IRAK4, suppresses the production of proinflammatory cytokines and proposed a model whereby IRAK-M prevents the dissociation of the IRAK1/IRAK4/MyD88 complex to inhibit downstream NF*κ*B activation [42]. Likewise, the splice variant IRAK1c acts in a similar manner by interacting with MyD88 and IRAK4. Like IRAK-M, IRAK1c cannot be phosphorylated by IRAK4 and thereby inhibits the dissociation of the complex, preventing activation of NF*κ*B [43]. Two other inhibitors have been identified downstream of the IRAK kinases: tumor necrosis factor-*α*-induced protein 3 (TNFAIP3, or A20) and IRF4. A20 is a ubiquitin-editing enzyme that deubiquitinates TRAF6 to inhibit the release of NF*κ*B from I*κ*B, thus inhibiting subsequent NF*κ*B-mediated gene transcription [3, 44]. IRF4 inhibits TLR signaling by competing for the same binding site on MyD88 with IRF5 [45]. IRF5 engages MyD88 downstream of TLR stimulation in order to promote production of proinflammatory cytokines. By IRF4 competing with IRF5 for binding, it inhibits the activation of IRF5 thus impairing IRF5-mediated cytokine production. 

## 3. TLRs in Autoimmunity

We know that TLRs are involved in protective immunity to invading microorganisms, but what are the consequences of misregulated TLR activation—meaning situations in which the TLR pathways are turned on too easily or activated by self-stimulus? The work of several groups suggests that in such instances, autoimmunity ensues. The resulting phenotype is characterized by the production of autoantibodies and tissue destruction. TLRs have been identified as instigators in type I diabetes, rheumatoid arthritis, and systemic lupus erythematosus. 

The nucleic acid binding TLRs are particularly implicated in SLE, an exceedingly complex and variable disorder. SLE is a systemic autoimmune disease characterized most commonly by antinuclear antibodies (ANAs). This disease is believed to affect at least 5 million individuals worldwide. Modern, effective treatment options are lacking for SLE, in that the primary treatment methods are currently corticosteroids, antimalarial, or anti-inflammatory drugs. There has not been an FDA-approved lupus treatment in over 40 years. The issue with this is the lack of identification of a “common denominator,” so to speak, for all lupus patients, which relates back to the complexity of the disease. However, the relatively new idea that Toll-like receptor pathways play crucial roles in lupus pathogenesis shows promise for potential drug targets. 

The role of nucleic acid binding TLR 7 has become quite apparent in both animal models of the disease and human patients. This receptor promotes autoantibodies and cytokines responsible for chronic inflammation [14, 46, 47]. One particularly important animal model at the forefront of these observations has been the BXSB mouse. This model was derived from a cross between C57BL/6 and SB/Le inbred strains, which resulted in male mice expressing an accelerated, lupus-like autoimmune disease phenotype characterized by production of ANAs and circulating immune complexes causing severe glomerulonephritis [48, 49]. Several subsequent studies have shown that the reason for the male bias in these mice was due to an X-to-Y chromosomal translocation of a gene cluster known as Y autoimmune accelerator (*Yaa*) and that the primary contributor to this accelerated autoimmunity is TLR 7 overexpression [16, 17]. Fc*γ*RIIB^−/−^ mice have a characteristic lupus-like phenotype with autoantibodies directed towards chromatin. However, Fc*γ*RIIB^−/−^ mice that also expressed *Yaa* exhibited a shift in autoantibody specificity from an antichromatin to an antinucleolar ANA pattern, consistent with an observed shift from chromatin to RNA-binding specificities [50]. This work further indicates a role for TLRs, in particular TLR 7, in the development of a specific autoimmune phenotype. Other groups have also described nucleic acid-binding TLR involvement in the production of autoantibodies. In one case in particular, Kono and colleagues ablated TLR 3, 7, and 9 signaling in B6-*Fas^lpr^* and BXSB mouse models and showed that these mice expressed decreases in autoantibody production [51]. 

Additional work has shown that defective apoptotic cell clearance is common among SLE patients, and this leads to development of antinuclear antibodies [52–54]. Our laboratory hypothesized that inefficient clearance of apoptotic debris triggers nucleic acid-binding toll-like receptors, which confer the B-cell response and subsequent ANA production. Injection of syngeneic late apoptotic thymocytes into wild type B6 mice led to anti-dsDNA and antihistone antibody production whereas injection into MyD88^−/−^ mice had no effect, suggesting that TLR stimulation is important in development of anti-dsDNA antibodies in situations of late apoptotic cell excess. Further studies using TLR 7- and TLR 9-deficient recipients of late apoptotic thymocytes showed that TLR 9 had no bearing on the development of anti-dsDNA and antihistone antibodies in this model, but TLR 7 did [55]. Moreover, the evidence suggested that TLR 7 promoted deposition of immune complexes in the renal glomeruli of these mice, possibly by influencing antichromatin antibody isotype. These studies suggest an important role for TLR 7 in the development of autoreactive antibodies and promotion of early events leading renal pathogenesis. 

The DNA-binding TLR 9 has also been heavily studied in connection with murine lupus. TLR 9 deficiency in some lupus models including MRL*^lpr/lpr^* mice can variably lead to reductions or alterations in antichromatin antibodies; however, TLR 9 deficiency paradoxically leads to disease exacerbation in multiple models [13, 14, 56–58]. In contrast, TLR3 deficiency failed to modify disease in MRL*^lpr/lpr^* mice [14]. 

Considering endogenous self-stimuli, then, murine studies indicate a central pathogenic role for TLR 7 in lupus pathogenesis and a complex overall protective role for TLR 9. Although endogenously triggered TLR 3 does not appear to drive lupus in the murine models investigated thus far, this particular TLR may play important roles in the promotion of lupus by environmental stimuli such as certain viral infections [59]. 

Most importantly, in addition to studies using mouse models, connections between TLRs and human lupus have also been identified. One of the most striking connections is the presence of elevated IFN-*α* as well as a Type I IFN gene signature in lupus patients [60, 61]. IFN-*α* is a key player in disease progression and severity and has even been shown to induce the production of autoantibodies when administered to nonautoimmune patients [62]. Another interesting finding was remission of SLE in a patient, which was attributed to unresponsiveness to both TLR 7 and 9 stimulation after development of common variable immunodeficiency- (CVID-) like disease [63]. Several groups have identified SNPs in the TLR 9 gene but have discovered that there is no correlation between these polymorphisms and SLE [64–67]. Although there was no correlation with particular SNPs, other groups showed that there was an upregulation of TLR 9 expression in B cells of lupus patients lending credence to the idea that TLR 9 could be involved in autoantibody production [68–70]. This finding led to the notion that the signaling proteins may be at fault in disease. Recent advances in genetic analysis techniques have allowed for the identification of a large number of genes (more than 25) that are associated with human lupus. At least three of the lupus-associated genes are involved in TLR signaling, including *IRF5*, *IRAK1, *and *TNFAIP3* [71–74]. The implication of these genes in lupus patients further indicates a role of TLRs in the disease phenotype. Whether enhanced stimulation of the TLRs or genetic factors altering the signaling cascade are at fault, the accumulation of evidence implies that TLR pathways do, in fact, play a role in the pathogenic process of SLE and remain excellent candidates for therapeutic targets to alleviate disease. 

Although most studies related to Toll-like receptors in SLE have focused primarily on the nucleic acid-binding TLRs, there is still a potential role for other TLRs in autoimmune disease. Komatsuda et al. looked at mRNA expression of several TLRs in peripheral blood mononuclear cells (PBMCs) from SLE patients. These studies indicated a slight increase in mRNA of TLR2, 7, and 9 in the lupus patients compared to healthy controls [75]. Additionally, others have reported an important role for TLR2 and 4 in the production of autoantibodies in the B6*^lpr/lpr^* autoimmune mouse model [76]. In these studies, TLR2- and TLR4-deficient B6*^lpr/lpr^*mice expressed lower titers of autoantibodies, excluding those directed towards nucleosome proteins. This shows that other TLRs could be candidates for targeted therapy in autoimmunity as well. 

## 4. TLRs as Therapeutic Targets

Significant evidence supports the involvement of TLRs in multiple disease processes, including Type I diabetes, RA, and SLE. The assertion that TLRs play a role in disease pathogenesis, with lupus in particular, indicates potential for therapeutic intervention by targeting these molecules or their signaling pathways. Since there are several proteins involved in TLR signaling, there are a number of targets that may be utilized for potential drugs. Some of the possibilities include, but are not limited to, development of TLR antagonists, inhibitors of downstream signaling events, activation of natural inhibitory molecules, or blockade of the effector molecules produced. Here we will discuss reports that highlight the potential usefulness of these different types of drugs.

The development of TLR antagonists for the nucleic acid-binding TLRs has proven to be a tedious process due to the similarity between eukaryotic and noneukaryotic nucleic acids. Despite this difficulty, Barrat and colleagues have developed immunoregulatory DNA sequences (IRS) that can bind TLR 9 but inhibit its activation and downstream effects. They have shown that these inhibitory ODNs can relieve inflammation in multiple scenarios. In one instance, mice injected with immunostimulatory sequences (ISSs) and D-galactosamine developed severe inflammation and died within days. However, when coinjected with the IRS, inflammation was decreased and mouse survival was prolonged [77]. Similar experiments demonstrated the same effect with TLR 7 as well. In addition to these studies, the same group developed a dual TLR 7/9 inhibitor. This oligodeoxynucleotide (ODN) was sufficient for inhibition of both TLR 7 and 9 signaling and protection against inflammation. These IRS sequences were also demonstrated to inhibit the production of IFN-*α* by human plasmacytoid dendritic cells (pDC), indicating the effectiveness of these inhibitors in human cells [78]. Because of the effectiveness in inhibiting TLR function, Barrat et al. also investigated the capacity of their ODNs to treat a lupus-prone mouse model. Several studies had previously established a potential role of IFN-*α* in the progression of autoimmunity in (NZB × NZW) F1 mice [79, 80]. Since IFN-*α* appears to play a central role in human SLE [81], this strain was selected as an ideal model for the application of IRS. IRS injections twice weekly in (NZB × NZW) F1 mice resulted in decreased ANAs, reduced glomerulonephritis at nine months, and increased rate of survival among the treated mice compared to untreated controls [82]. 

In addition to the studies by Barrat, two other groups used other inhibitory ODNs for targeting TLR signaling in autoimmunity. The ODNs designed by Dong et al. were injected into (NZB × NZW) F1 mice that were subsequently analyzed for kidney function and autoantibody production characteristic of lupus. Their results suggested a similar capacity of these inhibitory ODNs to minimize glomerulonephritis and reduce autoantibodies directed towards DNA [83]. Pawar et al. used IRS sequences as TLR 7 and 9 inhibitors in MRL*^lpr/lpr^* mice and showed a reduction in inflammatory cytokine levels and autoantibody titers, as well as a decrease in resulting tissue damage [84]. These experiments using inhibitory ODNs in treating lupus-prone animal models suggest strong potential for treatment of human lupus and other autoimmune diseases by targeting TLRs. 

Other potential therapeutic targets include the downstream signaling proteins involved in the TLR signaling cascade. There are a number of potential targets for this intervention including MyD88, TRAF6, and IRAK1 and 4. MyD88 is the common adaptor protein in most TLR signaling [85]. Since both TLR 7 and 9 utilize this protein, it is an excellent target to decrease the signaling that takes place in lupus. Two independent groups have shown that inhibiting MyD88 impairs the phosphorylation/activation of downstream kinases [86] and NF*κ*B activity [87]. Bartfai and colleagues developed a chemical mimic to the three amino acid sequence at the conserved BB-loop of the TIR domain of MyD88. The results of this mimic showed an inhibition of MyD88 and IL-1RI interaction by ablating phosphorylation of MAP kinases [86]. Loiarro et al. describe a similar mimic; however, they employ a peptide sequence instead of a chemical mimic. This inhibitor showed similar inhibition of MyD88 signaling indicated by impaired NF*κ*B activity [87]. They also went on to show that not only do these peptides inhibit dimerization of MyD88, but also inhibit the recruitment of IRAK1 and IRAK4 [88]. In view of the fact that MyD88 is a central mediator in TLR 7 and 9 signaling, these inhibitors of MyD88 dimerization and recruitment of essential kinases could prove to be an effective treatment option in lupus by abolishing the aberrant TLR signaling that induces the elevated type I IFN levels and persistent inflammation. 

Another potential target for minimizing damaging TLR signaling is IRAK4. Briefly, IRAK4 is a kinase that interacts with MyD88 and TRAF6 and is associated with the activation of the downstream transcription factors in TLR signaling. Based on IRAK4-deficient animal models, this kinase has been shown to be indispensible in TLR signaling [89, 90]. Because of this idea that IRAK4 is necessary for production of effectors in a TLR-dependent manner, it has been subjected to targeted therapy. However, IRAK4 inhibitors developed to date have yet to be analyzed for efficacy in animal models [91–94]. 

The last actively studied therapeutic approach we will discuss targets the end-product effectors of TLR signaling as opposed to the signaling cascade itself. An important target that is now accepted to be characteristic of SLE is IFN-*α*. This cytokine is found in elevated levels among lupus patients, and high levels associate with worsened measures of disease activity [81]. Due to the apparent pathogenic nature of IFN-*α*, inhibitory monoclonal antibodies have been developed against the cytokine as a treatment for SLE [95]. One such drug is currently in phase II clinical trials and has shown promise thus far in relieving symptoms of lupus [96]. Among the TLR and TLR-related targets for therapy in lupus, this antibody directed towards IFN-*α* appears to be the most outstanding thus far.

Currently, active research on this topic is concerned with negatively regulating molecules that activate TLR-dependent signaling, but what about activating some of the natural inhibitory proteins, such as Triad3A, MyD88s, SHP-1, IRAK-M, IRAK1c, IRF4, and A20? These naturally occurring inhibitors of the signaling cascade could be targeted for agonistic drugs. Xie et al. reported that IRAK-M-deficient mice injected with tumor cells develop increased CD4^+^ T-cell differentiation and upregulation of costimulatory molecules and other activation markers on B cells, compared to wild-type controls, indicating an inhibitory role for IRAK-M [97]. Although IRAK-M deficiency appears to have a positive role in cancer models, the inverse is likely to be true for autoimmune situations. Stimulation of production or activity of IRAK-M may provide another alternative therapy in SLE. Similarly, promoting the production or activity of the other endogenous TLR signaling inhibitors, Triad3A, MyD88s, SHP-1, IRAK1c, IRF4, or A20, could provide additional options for effective treatment for systemic autoimmunity. Utilizing these endogenous proteins could provide a new avenue to decrease TLR-mediated inflammation in patients with autoimmune diseases, with each of these targets allowing a different route to decreasing production of inflammatory cytokines and subsequent risk of tissue damage. 

Although the approaches presented here are in the context of nucleic acid-binding TLRs, the proposed strategies for TLR signaling inhibition could actually act more globally. Since most TLRs can signal through the adaptor protein MyD88, these potential therapeutic targets could prevent signaling through all TLRs with the exception of TLR 3. The specific TLR 7 and TLR 9 inhibitors discussed earlier would be ideal as a treatment method to avoid a total inhibition of the first line of microbial defense; however, with evidence revealing potential roles for other Toll-like receptors, namely, TLR 2 and 4, in SLE, a more widespread inhibition may be an important aspect worth investigating. 

## 5. Conclusions

Although TLRs are extraordinarily important in pathogen recognition and normal immune function, including that of both innate and adaptive arms of the immune system, they have also been ascribed causative roles in autoimmunity. Various studies ranging from lupus-prone mouse models, to genome-wide association studies in human lupus patients, to *in vitro* studies with patient cells have indicated a significant role for nucleic acid-binding TLRs in the progression and severity of lupus and other autoimmune diseases. From these studies, we have learned about the involvement of TLRs in lupus-prone mouse models, the association of polymorphisms in *IRF5, IRAK1, *and *TNFAIP3* with human disease, and the upregulation of TLRs in SLE patients. Due to the accumulation of these data suggesting a detrimental role of TLR signaling in lupus, efforts are currently underway to ascertain reliable treatment methods based on the targeting of TLRs and their signaling counterparts. Inhibition of TLRs and their signaling pathways have proven to be efficacious in lupus-prone mouse models and successfully inhibit IFN-*α* production by human pDC *in vitro*. Although the direct TLR antagonists have not been studied in human patients, inhibitors of IFN-*α*, a primary downstream effector of TLR signaling and important disease mediator in SLE, have been developed and are currently undergoing clinical trials. Monoclonal antibody treatments have been successfully utilized in the setting of rheumatic diseases for some time now and are likely to comprise a new armament the realm of lupus treatment. Although there has not been a new drug approved for the treatment of lupus in many years, current investigation regarding the targeting of TLRs and their downstream effectors is showing some promise and warrants high expectations.
